# Selective ablation of P53 in pancreatic beta cells fails to ameliorate glucose metabolism in genetic, dietary and pharmacological models of diabetes mellitus

**DOI:** 10.1016/j.molmet.2022.101650

**Published:** 2022-12-05

**Authors:** Celina Uhlemeyer, Nadine Müller, Michael Rieck, Jennifer Kuboth, Caroline Schlegel, Kerstin Grieß, Tim Florian Dorweiler, Sonja Heiduschka, Jürgen Eckel, Michael Roden, Eckhard Lammert, Markus Stoffel, Bengt-Frederik Belgardt

**Affiliations:** 1 Institute for Vascular and Islet Cell Biology, German Diabetes Center, Leibniz Center for Diabetes Research at Heinrich-Heine University, Düsseldorf, Germany; 2 German Center for Diabetes Research (DZD e.V.), Neuherberg, Germany; 3 Institute for Clinical Diabetology, German Diabetes Center, Leibniz Center for Diabetes, Düsseldorf, Germany; 4 Department of Endocrinology and Diabetology, Medical Faculty and University Hospital Düsseldorf, Heinrich Heine University, Düsseldorf, Germany; 5 Institute of Metabolic Physiology, Heinrich Heine University, Düsseldorf, Germany; 6 Institute of Molecular Health Sciences (IMHS), ETH Zürich, Zürich, Switzerland; Competence Center Personalized Medicine, ETH Zürich, Zürich, Switzerland; Medical Faculty, University of Zürich, Zürich, Switzerland

**Keywords:** Pancreatic beta cell, Apoptosis, Type 1 diabetes, Type 2 diabetes

## Abstract

**Objective:**

Beta cell dysfunction and death are critical steps in the development of both type 1 and type 2 diabetes (T1D and T2D), but the underlying mechanisms are incompletely understood. Activation of the essential tumor suppressor and transcription factor P53 (also known as TP53 and Trp53 in mice) was linked to beta cell death *in vitro* and has been reported in several diabetes mouse models and beta cells of humans with T2D. In this article, we set out to determine the beta cell specific role of P53 in beta cell dysfunction, cell death and development of diabetes *in vivo*.

**Methods:**

We generated beta cell specific P53 knockout (P53^BKO^) mice and used complementary genetic, dietary and pharmacological models of glucose intolerance, beta cell dysfunction and diabetes development to evaluate the functional role of P53 selectively in beta cells. We further analyzed the effect of P53 ablation on beta cell survival in isolated pancreatic islets exposed to diabetogenic stress inducers *ex vivo* by flow cytometry.

**Results:**

Beta cell specific ablation of P53/Trp53 failed to ameliorate glucose tolerance, insulin secretion or to increase beta cell numbers in genetic, dietary and pharmacological models of diabetes. Additionally, loss of P53 in beta cells did not protect against streptozotocin (STZ) induced hyperglycemia and beta cell death, although STZ-induced activation of classical pro-apoptotic P53 target genes was significantly reduced in P53^BKO^ mice. In contrast, Olaparib mediated PARP1 inhibition protected against acute *ex vivo* STZ-induced beta cell death and islet destruction.

**Conclusions:**

Our study reveals that ablation of P53 specifically in beta cells is unexpectedly unable to attenuate beta cell failure and death *in vivo* and *ex vivo*. While during development and progression of diabetes, P53 and P53-regulated pathways are activated, our study suggests that P53 signaling is not essential for loss of beta cells or beta cell dysfunction. P53 in other cell types and organs may predominantly regulate systemic glucose homeostasis.

## Highlights

• Beta cell specific P53 ablation cannot attenuate beta cell failure in diabetes models.
• P53 signaling is activated but not essential during beta cell demise.
• PARP1 inhibition but not P53 ablation protects against DNA-damage induced beta cell death.

## 1 Introduction

Diabetes mellitus affects more than 500 million people worldwide [1]. The main characteristics of diabetes are insufficient insulin secretion and/or disturbed cellular insulin signaling (insulin resistance), resulting in chronic hyperglycemia [2]. Type 1 diabetes (T1D), affecting approx. 5–10% of all persons with diabetes, is an autoimmune disease causing loss of beta cells and manifests mainly but not exclusively in childhood and adolescence. Approximately 90% of people with diabetes are diagnosed with type 2 diabetes (T2D), a multifactorial disease with obesity, age and genetic predisposition as main risk factors. In T2D, the extent and onset of insulin resistance and beta cell dysfunction appears to be variable, potentially in line with its recently proposed subtypes [[2], [3], [4], [5]]. *Post mortem* analyses of pancreata from people with T2D have detected varying levels of beta cell death and reduced beta cell mass [6,7], and multiple animal models of T2D show massive beta cell apoptosis [[8], [9], [10], [11]]. On the other hand, processes like beta cell trans- or dedifferentiation, as well as loss of beta cell function have also been linked to T2D development [5,12,13]. Beta cells are known to experience multiple types of cellular stress during diabetes development, including but not limited to endoplasmic reticulum stress, inflammation, oxidative stress, glucolipotoxicity and DNA damage [2,14]. The transcription factor tumor protein P53 (P53, also known as transformation related protein TP53, or Trp53 in rodents) is essential for tumor prevention and regulation of cell survival and death [15,16], but also plays pleiotropic functions in cell biology and physiology [17]. Several animal and human studies link P53 activity to diabetes progression. For instance, we previously found expression of canonical P53 target genes to be upregulated in islets of obese and diabetic *db/db* mice and linked P53 activity to increased abundance of the pro-diabetogenic microRNA-200 family [8]. Moreover, beta cell specific ablation of P53 protected against beta cell death (but not dysfunction) induced by a rare disease-inducing glucokinase mutation [18]. In addition, whole body P53 knockout (KO) mice (after bone marrow transplantation from wildtype mice) showed increased mitophagy, resulting in maintained insulin secretion and glucose tolerance in mice with chemically induced beta cell destruction [19]. Accordingly, beta cell specific ablation of Ataxia Telangiectasia Mutated (ATM), one of several upstream regulators of P53, ameliorated chemically induced hyperglycemia [20]. In contrast, P53 action was also reported to have beneficial effects in diabetic mouse models. Secchiero et al. showed that systemic P53 de-inhibition and stabilization by treatment with the small molecule Nutlin-3 reduced streptozotocin (STZ)-induced hyperglycemia, in part by acting on the immune system [21]. To summarize, most studies strongly indicate that inhibition or depletion of P53 can attenuate diabetes development potentially by preserving beta cell function and survival. However, as most of these studies were conducted with either conventional P53 KO mice or systemic P53 manipulation by virus administration or pharmacological treatments, the beta cell autonomous role of P53 in development and progression of diabetes is incompletely understood. To unravel the role of this essential protein in cellular physiology, we generated mice with beta cell specific ablation of P53 and analyzed this strain in genetic, dietary and pharmacological models of glucose intolerance, beta cell dysfunction and diabetes development.

## 2 Material and methods

### 2.1 Experimental animals

All experiments were approved by the Ethics Committee of the State Ministry of Agriculture, Nutrition and Forestry (State of North Rhine-Westphalia, Germany). The Ins1-Cre mice [22], conditional TP53 mice (also known as Trp53 mice) [23], conditional ATM mice (backcrossed onto a C57BL/6JRj background (Janvier) for at least 7 generations) [24], conventional PDX1 mice [25], and tdTomato reporter mice [26] on a C57BL/6J background have been described before. For analyses in dietary and pharmacological models of diabetes, we generated beta cell specific P53 KO mice (hence denoted as P53^BKO^) by cross-breeding mice carrying the Ins1-Cre transgene with P53 floxed mice (#008462, Jackson Laboratories, USA). For dietary and pharmacological interventions, control mice were heterozygous for the Ins1-Cre transgene but carried two wildtype P53 alleles (Ctrl). For analysis of P53 function on a background of heterozygous PDX1 deficiency, we generated mice lacking one PDX1 allele and at the same time lacking P53 specifically in beta cells (genotype Ins1-Cre^tg/wt^, P53 ^fl/fl^, PDX1^wt/KO^, denoted as PDX1^wt/KO^ P53^BKO^) by cross-breeding P53^BKO^ mice with PDX1 heterozygous mice. Resulting littermates not carrying the Cre allele, but homozygous for a loxP flanked P53 allele and heterozygous for the PDX1 null allele were used as control for this genetic intervention (PDX1^wt/KO^ Ctrl). As second control, we also used P53^BKO^ mice carrying two wildtype PDX1 alleles (PDX1^wt/wt^ P53^BKO^). For flow cytometric analyses, we cross-bred Rosa26-tdTomato reporter mice with P53^BKO^ (Tomato:P53^BKO^), ATM^BKO^ (Tomato:ATM^BKO^) or Ins1-Cre transgenic mice (genotype Ins1-Cre^tg/wt^, Rosa26-tdTomato^fl/wt^ or Ins1-Cre^tg/wt^, Rosa26-tdTomato^fl/fl^, denoted as Tomato^Beta^), resulting in a beta cell specific red fluorescence signal. For qPCR analyses of sorted beta and non-beta cells, also PDX1 wildtype and heterozygous mice with or without additional beta cell specific KO of P53 were cross-bred with Rosa26-tdTomato reporter mice (groups additionally denoted as Tomato reporter strains). All Tomato reporter mice were heterozygous for the Ins1-Cre knock-in allele. Male mice were used for all studies due to the inherent resistance of beta cells of female (C57BL/6J) mice against beta cell dysfunction, beta cell death and diabetes [27]. An overview of all used diabetes mouse models and their different properties is provided in Table S1. Mice were housed at three to six mice per cage (Macrolon type III) at a constant temperature of 22C° and a 12 h light–dark cycle (lights on at 6 AM). Animals had free access to food and water ad libitum. After weaning at the age of 21–28 days, mice were fed either a standard laboratory chow or a high fat diet (HFD) containing 45 kcal% fat, 20 kcal% protein, and 35 kcal% carbohydrates with 4.73 kcal/g energy (D12451; Research Diets, New Brunswick, NJ) until the end of the respective study. For flow cytometric analysis of islet cell viability or gene expression analysis of pancreatic islets, age-matched mice were sacrificed at the age of 2–11 months.

### 2.2 Genotyping

Genotyping was performed using standard protocols using appropriate primers (Table S2) and GoTaq G2 Hot Start Green Master Mix (#M7423, Promega).

### 2.3 Body weight, blood glucose levels, glucose and insulin tolerance tests

Body weight and blood glucose levels were determined weekly (unless stated otherwise) with an electronic scale and Contour XT glucometer (Bayer Consumer Care AG, Leverkusen, Germany), respectively. For intraperitoneal (i.p.) glucose tolerance tests (ipGTT), 16 h fasted mice were injected i.p. with 0.75–2 g/kg body weight d-glucose in PBS (as stated in each subfigure or figure legend). Glucose bolus was chosen dependent on expected maximal glucose excursions. Blood glucose levels were measured every 20 min and blood was collected before (0 min), as well as 20 and 120 min after glucose injection in heparin-coated tubes. After centrifugation, plasma was stored at −20 °C (short-term <1 week) or −80 °C (long-term >1 week). Non-responding mice (BG increase <20% at 20 min, potentially due to incomplete i.p. injection) were excluded from analysis (1 mouse each in Figs. S4E and S4F). For i.p. insulin tolerance tests (ipITT), mice were fasted for 4 h and then injected i.p. with 0.75 U/kg body weight insulin (Insuman Rapid, Sanofi) diluted in PBS. Blood glucose levels were measured every 20 min for 2 h.

### 2.4 Analysis of plasma insulin

Insulin levels were measured by ELISA (Ultra Sensitive Rat Insulin ELISA Kit; #90060, CrystalChem) according to the manufacturer's instructions in 5 μl plasma collected during ipGTTs. Mice with hemolytic plasma samples or insulin values below detection limit were excluded from analysis (2 mice in Fig. S4I).

### 2.5 Administration of STZ

At week 12 of age, 6 h fasted mice of MLD-STZ or HFD-STZ cohorts were injected i.p. with 40 mg/kg STZ at 5 consecutive days (MLD-STZ model) or once with 150 mg/kg (HFD-STZ model). STZ was stored at −20 °C and reconstituted immediately before injection in citrate buffer. One MLD-STZ cohort (both Ctrl and P53^BKO^ mice) did not respond to STZ injections (no increase in BG levels), potentially due to temperature fluctuations during STZ transport, and was excluded from analysis.

### 2.6 Isolation, dispersion and culture of pancreatic islets

Mice were sacrificed by cervical dislocation and the pancreatic islets were isolated by ductal liberase TL (#5401020001, Merck) perfusion of the pancreas, based on a previously described method with modifications [8]. In short, pancreas was dissected and incubated for 17 min at 37 °C in a shaking waterbath. Digestion was stopped with 40 ml of 10% Fetal Calf Serum (FCS, Gibco) in RPMI, followed by moderate shaking for 30 s. Cells were spun down at 300 × g for 5 min, resuspended in 10 ml RPMI, strained through a sieve, filled up to 50 ml with RPMI and spun down again. Resulting pellet was resuspended in 3 ml 1.119 g/ml Histopaque (#11191, Merck) and overlayed with 1.083 and 1.077 g/ml Histopaque (#10831 and #10771, Merck) and RPMI, 2.5 ml each. After density gradient centrifugation at 935 × g for 20 min with acceleration and brake active, but at lowest setting, islets accumulated between the two upper phases, were transferred and washed with RPMI containing 10% FCS. After centrifugation, islets were transferred into petri dishes containing 10 ml full islet medium (DMEM with 10% FCS, 11.11 mM glucose, 2 mM glutamax, 1 mM sodium pyruvate, 11.2 mM HEPES, 0.175 mM beta-mercaptoethanol and 100 U/ml penicillin/streptomycin) and incubated at 37 °C with 5% CO_2_. Islets were harvested 3–72 h post isolation, depending on the experimental setup. For staining of dispersed islet cells (Fig. S3L-P), islets were transferred into 15 ml tubes, washed with 10 ml PBS and trypsinized with 3 ml 0.05% Trypsin/EDTA (#25300-054, Thermo Fisher) for 12 min with additional mechanical disruption by carefully pipetting up and down three times in between. Trypsinization was stopped by adding 10 ml FCS containing islet medium. Cells were spun down at 300 × g for 5 min, supernatant was discarded and cells were resuspended in 1 ml medium. Cells were counted and seeded in a 96-well-plate (20.000 cells/well in 100 μl each) suitable for microscopy. Cells were spun down at 300 × g for 3 min to support cell adhesion, incubated for 6 h at 37 °C, 5% CO_2_ and fixed by adding 100 μl 4% PFA for 20 min at RT. Cells were washed with PBS and used for immunostaining (performed as described in 2.10 with downscaled volumes) and finally imaged at 40× magnification using a laser scanning microscope (LSM 880, Zeiss) in confocal mode.

### 2.7 RNA isolation and quantification

Islets were collected 3–24 h after isolation and RNA was isolated using Trizol reagent (#30-2020, VWR) or RNAeasy Plus Mini Kit (#74136, Qiagen) according to the manufacturer's protocols. RNA was reverse transcribed using High-Capacity cDNA Reverse Transcription Kit (#4368813, Thermo Fisher). Quantitative realtime PCR was performed in a Quantstudio 7 (Applied Biosystems) by using gene specific primers (Table S2) and Perfecta Sybr Green FastMix (#733-1391, VWR) or Luna® Universal qPCR Master Mix (#M3003, NEB). Ct values were normalized to *36B4* and *GUSB* mRNA levels.

### 2.8 Immunoblotting

A minimum of 50 islets was collected in 1.5 ml tubes, spun down for 5 min at 300 × g, washed twice with PBS and the resulting pellet lysed in Bio-Plex buffer (30 μl per 50 islets, #171304011, Bio-Rad). 4× Laemmli buffer (#1610747, Bio-Rad) with DTT was added to samples, and after boiling for 5 min, 10 μl of protein samples were separated by SDS-PAGE using pre-cast 4–15% Stainfree gels (#4568086, Bio-Rad; allowing detection of tryptophan-containing proteins using a proprietary trihalo compound), transferred by electroblotting and PVDF membranes blocked in TBS with 0.1% Tween-20 (TBST) containing 5% nonfat-dried milk or BSA for 1 h. Membranes were incubated with primary antibodies (see 2.13) overnight at 4 °C, washed thrice with TBST for 10 min, exposed to HRP-conjugated secondary antibodies for 1 h at room temperature (RT), washed thrice again with TBST for 10 min and developed using ECL Western Blotting Substrate (#170-5061, Bio-Rad). Quantification of all bands was performed using Image Lab software (Bio-Rad). The mean value of relevant control islets was set to 1 and all other values normalized to that.

### 2.9 Chemicals and diabetogenic treatments

Islets were treated with: essentially fatty acid free bovine serum albumine (BSA, #A8806, Merck); a cytokine mix (for 40 h) composed of tumor necrosis factor alpha (TNFα, 1000 U/ml, #PMC3014, Thermo Fisher), interleukin 1 beta (IL1β, 50 U/ml, #Phc0814, Thermo Fisher) and interferon gamma (IFNγ, 1000 U/ml for 40 h, #PMC4031, Thermo Fisher); Nutlin-3 (10 μM for 6 h, #N6287, Sigma-Aldrich); Olaparib (10 μM for 3 h + 16 h (pre- and co-treatment with STZ), #S1060, Selleckchem); or streptozotocin (STZ, 0.5, 1.0 or 1.5 mM for 6 or 16 h, #S0130, Sigma-Aldrich, as stated in each figure legend). Due to its reported instability in solution, STZ powder was kept at −20 °C at all times and was always diluted immediately before treatment.

### 2.10 Immunohistochemistry

Pancreata were fixed in 4% PFA o/n and dehydrated by transferring into 7.5, 15 and 30% Sucrose in PBS (at least 6 h each), before embedding in NEG-50 (#11912365, Fisher-Scientific). Of each pancreas, 12-18 10 μm sections with a minimum distance of 180 μm between each other were analyzed. Slides were washed for 5 min with PBS and PBS-T (PBS + 0.2% TritonX-100) before 1 h blocking with 5% BSA in PBS-T at RT in a humidified chamber and covered with parafilm. Directly labeled or unconjugated primary antibodies were added o/n at 4 °C (see 2.13). After washing with PBS twice and PBS-T once for 10 min each, secondary antibody (1:500) and Hoechst 33342 (1:5000) were added for 1 h at RT diluted in blocking buffer. Slides were washed thrice for 10 min with PBS. Fluoroshield (#F6182, Merck) was added for mounting with coverslips. Slides were imaged with a VS200 Slide Scanner (Olympus) using a 40× objective and quantification was performed with QuPath [28] in a blinded manner, using a custom analysis macro (available upon request) and trained pixel classifier for semi-automated islet area identification and cell counting (Fig. S3). In more detail, insulin, Nkx6.1 or glucagon positive areas with a minimum size of 40 μm^2^ were identified by the macro as islets. Islet were encircled automatically, but needed manual adjustments (separation of adjacent islets, deletion of islets with less than three Nkx6.1^positive^ cells, tightening of islet annotations). Next, Hoechst/Nkx6.1/Nkx6.1 and Ki67 (double-) positive cells were automatically identified (by threshold) and counted. The automatic detection of Ki67^positive^ cells was manually controlled to avoid false positive detections due to e.g. high background signal (e.g. observed in proximity to large vessels) and if necessary, manually corrected.

### 2.11 Flow cytometric cell death analysis

For flow cytometric analyses, islets of wildtype mice (C57Bl/6JRj, Janvier), Cre-negative colony mates, Tomato^Beta^ mice, Tomato:P53^BKO^ mice and Tomato:ATM^BKO^ mice were used, depending on the experiment. Islets (approx. 70-100) were treated in 6-well plates as a single technical replicate, based on low interexperimental variability. Treatments were started 24 h–48 h post isolation, and treatment durations were optimized to detect significant cell death (STZ 16 h, Cytokines 40 h). After treatment, the supernatant (containing single dead/dying cells) was transferred to FACS tubes. Residual islets were trypsinized (as described in 2.6) to achieve a single cell suspension, before transferring to the respective FACS tubes. Cells were spun down for 5 min at 300 × g followed by a wash step with PBS. Cells were incubated in 500 μl FVS520 (#564407, BD Biosciences, diluted 1:1000 in PBS) for 15 min at RT. After two wash steps with PBS, cells were resuspended in 300 μl PBS and the amount of FVS520^positive^ (dead) and FVS520^negative^ (living), as well as tdTomato^positive^ (beta) and tdTomato^negative^ (non-beta) cells was measured using a FACSCalibur (BD Biosciences) or CytoFLEX S (Beckman Coulter). Notably, no double positive population was detectable consistent with our observations that dying/dead cells lose the tdTomato signal, likely due to membrane barrier loss. Hence, percentage of total dead (FVS520^positive^) and living (FVS520^negative^) beta (tdTomato^positive^) vs non-beta (tdTomato^negative^) cells was quantified using FlowJo V10 software (BD Biosciences). Single stained control samples were used for compensation and to confirm that the FVS520 signal was reproducibly stronger compared to beta cell autofluorescence.

### 2.12 FACS of beta- and non-beta cells

For sorting of beta- and non-beta cells, 70-350 islets of Tomato reporter mice (genotype for Ins1 and Rosa26 locus: Ins1-Cre^tg/wt^ and Rosa26-tdTomato^fl/wt^ or Rosa26-tdTomato^fl/fl^) were trypsinized (as described in 2.6), resuspended in 300 μl islet medium, strained through a 70 μm sieve and sorted using a CytoFLEX SRT (Beckman Coulter). Gating strategy is depicted in Figure S8.

### 2.13 Antibodies

The following antibodies were used: rabbit anti pS139-H2A.X (#9718, Cell Signaling, 1:1000), rabbit anti LSD1 (#2184, Cell Signaling, 1:1000), rabbit anti P53 (#32532, Cell Signaling, 1:1000), rabbit anti pS/pT-ATM/ATR Substrate Motif (#6966, Cell Signaling, 1:1000), rabbit anti PDX1 (#5679, Cell Signaling, 1:1000), goat anti rabbit IgG –HRP conjugated (#401353, Merck, 1:5000), guinea pig anti insulin (#IR00261-2, Agilent, dilution 1:10), rat anti Ki67 (#14-5698-82, Thermo Fisher, dilution 1:750) directly labeled with CF568 (#MX568S100, Merck, final conc.: 0.5 mg/ml), goat anti Nkx6.1 (#AF5857, R&D Systems, dilution 1:300) directly labeled with CF647 (#MX647S100, Merck, final conc.: 0.2 mg/ml), rabbit anti glucagon (#ab92517, abcam, dilution 1:750) directly labeled with CF750 (#MX750S100, Merck, final conc.: 0.3 mg/ml) or in combination with donkey anti rabbit-AF488 (#A-21206, Thermo Fisher, dilution 1:500, Fig. S3M, P), and goat anti guinea pig-AF488 (#A-11073, Thermo Fisher, dilution 1:500).

### 2.14 Statistical analysis

Values are reported as individual values and/or mean ± standard error of the mean (SEM) showing each individual experiment (or mouse) as a single data point. The number of independent experiments and replications, or cohorts and mice is stated in each figure legend and also visible in the quantification figures. No statistical method was used to predetermine sample size, but instead was based on preliminary data and previous publications as well as observed effect sizes. Due to the small sample sizes, normality testing was not performed. Each statistical test is described in the figure legends. Depending on the number of variables, a regular one- or two-way analysis of variance (ANOVA), followed by Tukey's or Sidak's multiple comparison test was used. For enhanced clarity and reduced visual cluttering, not all comparisons are shown, as stated in the figure legends. For analyses of *in vivo* cohort data, statistical testing was only performed between different genotypes and not between different time points. Experiments with only two groups and one variable being tested were analyzed by an unpaired two-sided Student's t-test or by multiple t-test's without correction for multiple comparisons. Statistical analyses were performed using the Graphpad Prism (GraphPad Software, La Jolla, CA, USA, Version 9) software. p-values smaller than 0.05 are shown in each figure (except if stated otherwise) and are rounded to the third decimal place.

## 3 Results

### 3.1 HFD-feeding results in glucose intolerance independent of beta cell P53

**Figure 1:**
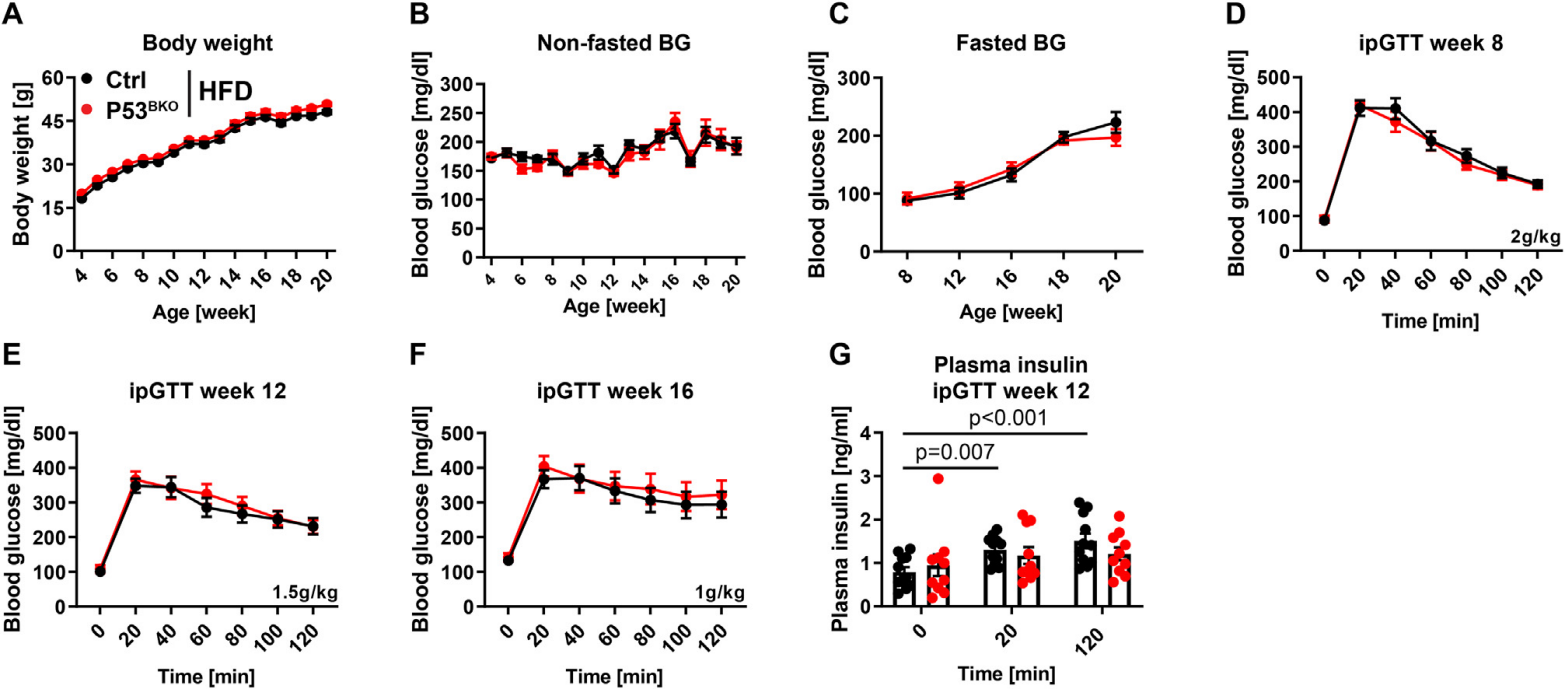
**Beta cell P53 does not alter glucose metabolism and insulin resistance upon HFD feeding**. (**A**) Body weight (BW), (**B**) non-fasted blood glucose (BG) levels and (**C**) fasted BG levels of Ctrl and P53^BKO^ mice fed a high fat diet (HFD) starting at week 4 of age. Fasting period was 16 h (week 8, 12, 16), 4 h (week 18) and 6 h (week 20). (**D**–**F**) Glucose excursion during an intraperitoneal glucose tolerance test (ipGTT) at different ages of mice described in (A–C). Glucose bolus is indicated for each ipGTT. (**G**) Plasma insulin levels at time points 0, 20 and 120 min during ipGTT described in (E). (**A-G**) Shown are mean values and (**G**) individual values ± SEM (n = 2 independent cohorts with 11 Ctrl vs. 10 P53^BKO^ mice in total). (**A**–**G**) Significance was determined by two-way ANOVA followed by Sidak's multiple comparison test.

Since P53 whole body KO mice develop early-onset cancer, immunodeficiency and show a massively reduced life span [29], we generated mice lacking P53 specifically in beta cells (P53^BKO^ mice). Recombination of the P53 gene locus as well as loss of P53 expression was verified on genomic DNA, mRNA and protein level in islet samples, as well as in fluorescence-activated cell sorting (FACS)-purified tdTomato^positive^ beta and tdTomato^negative^ non-beta cells, which confirmed near-total and specific ablation of P53 without any observable effect on beta cell marker genes such as *INS1/2* (Figs. S1A–H). We initially challenged P53^BKO^ and Ctrl mice with a high fat diet (HFD), an established model for low to moderate beta cell stress and early T2D development (Fig. S1I). While HFD feeding does not cause beta cell failure or overt diabetes, beta cells need to undergo expansion and hypersecrete insulin to maintain near-normal glucose levels in this model [30,31]. We did not detect any significant changes between HFD-fed Ctrl and P53^BKO^ mice in body weight gain, glucose tolerance, insulin secretion and sensitivity, as determined by glucose and insulin tolerance tests at several ages (Figure 1A–G and S1J, K). Thus, beta cell selective ablation of P53 does not affect insulin secretion or glucose metabolism when beta cells are under HFD-induced metabolic stress.

### 3.2 HFD-fed PDX1^wt/KO^ mice develop severe glucose intolerance independent of beta cell P53

**Figure 2:**
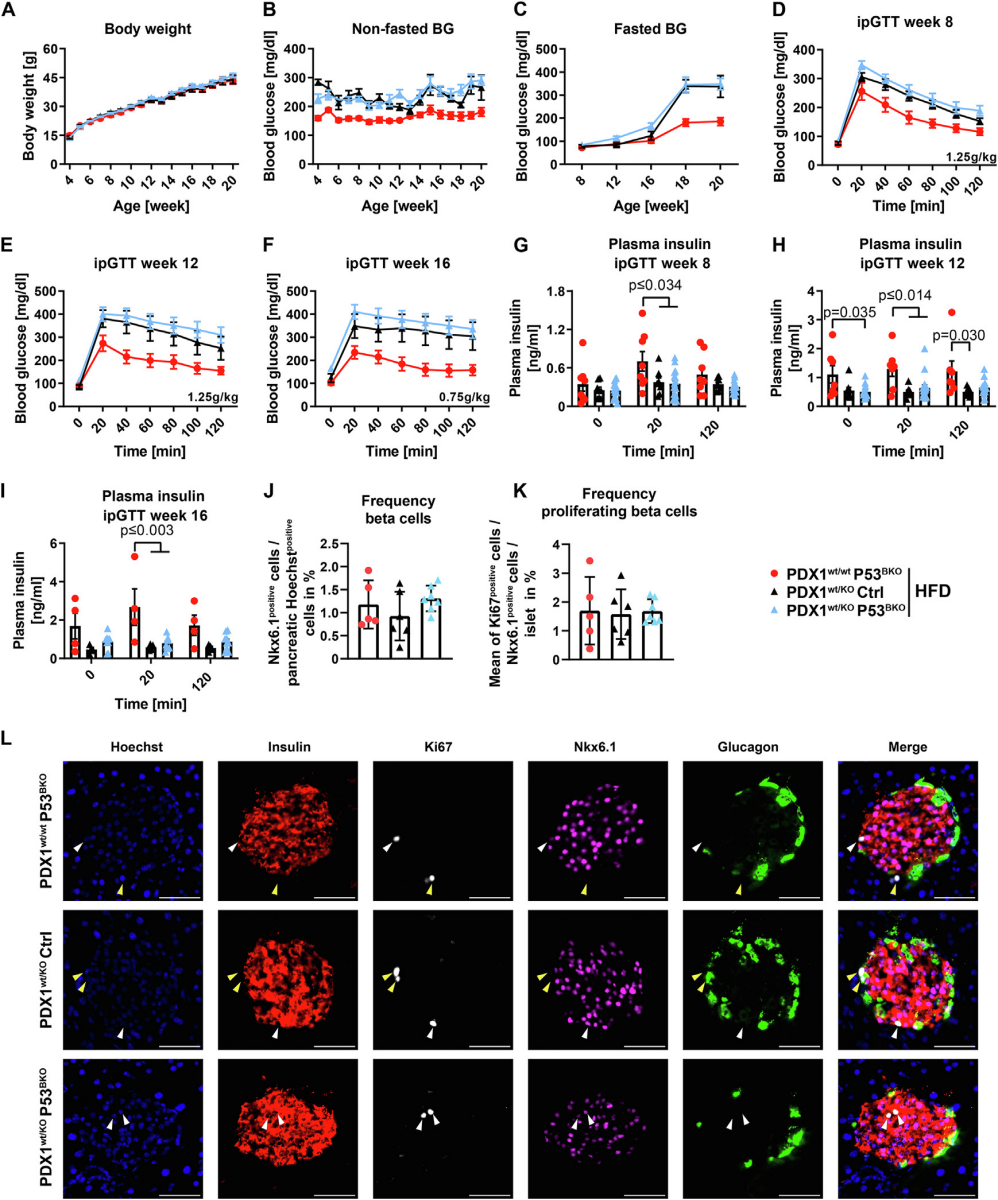
**HFD fed PDX1^wt/KO^ mice show severe glucose intolerance independent of beta cell P53**. (**A**) Body weight, (**B**) non-fasted blood glucose (BG) levels and (**C**) fasted BG levels of PDX1^wt/wt^ P53^BKO^, PDX1^wt/KO^ Ctrl and PDX1^wt/KO^ P53^BKO^ mice fed a high fat diet (HFD) starting at week 4 of age. Fasting period was 16 h (week 8, 12, 16), 4 h (week 18) and 6 h (week 20). (**D**–**F**) Blood glucose excursions during intraperitoneal glucose tolerance tests (ipGTTs) at different ages of mice described in (A–C). Glucose bolus is indicated for each ipGTT. (**G**–**I**) Plasma insulin levels at time points 0, 20 and 120 min during ipGTTs described in (D–F). (**A**–**I**) Shown are mean and (**G**–**I**) individual values ± SEM (n = 3 independent cohorts with 7 PDX1^wt/wt^ P53^BKO^ vs. 8 PDX1^wt/KO^ Ctrl vs. 17 PDX1^wt/KO^ P53^BKO^ mice in total). (**J**–**L**) Quantification and representative images of immunostainings detecting cell nuclei (Hoechst), insulin, proliferating cells (Ki67), beta cells (Nkx6.1) and glucagon in tissue sections of pancreata from mice described in (A–C) (for clear visibility, image contrast settings were identically optimized in all groups). White arrows indicate proliferating beta cells (Ki67^positive^ + Nkx6.1^positive^ cells) and yellow arrows proliferating non-beta cells (Ki67^positive^ + Nkx6.1^negative^ cells). Glucagon and insulin staining was used to identify islets and calculate islet area. (**J** + **K**) Quantification of (**J**) Nkx6.1^positive^ beta cells normalized to total cell numbers of pancreatic sections and (**K**) (Nkx6.1^positive^ + Ki67^positive^) proliferating beta cells per islet normalized to islet cell numbers of mice described in (A–C). Shown are mean and individual values ± SEM (n = 2 independent cohorts with 5 PDX1^wt/wt^ P53^BKO^ vs. 6 PDX1^wt/KO^ Ctrl vs. 7 PDX1^wt/KO^ P53^BKO^ mice in total. Of each mouse n = 12–18 pancreas sections and at least 4000 Nkx6.1^positive^ cells were analyzed). Significance was determined by (**A**–**I**) two-way or (**J** + **K**) one-way ANOVA followed by Sidak's or Tukey's multiple comparison test, respectively. Significant comparisons (**A**–**F**) vs PDX1^wt/wt^ P53^BKO^ or (**G**–**I**) between different time points are not shown to ensure readability.

We reasoned that potentially, beta cell stress induced by HFD was insufficient to provoke a P53-dependent phenotype, since HFD feeding decreased glucose tolerance, but did not lead to overt hyperglycemia. Hence, we aimed to challenge P53^BKO^ mice in a model with moderate to high beta cell stress. Pancreatic and Duodenal Homeobox 1 (PDX1) is a transcription factor that is essential for pancreas development and that postnatally regulates beta cell transcription and function in humans and mice. Importantly, rare cases of PDX1 heterozygosity lead to maturity-onset diabetes of the young 4 (MODY4) [32], and a partial reduction of PDX1 expression is a key observation in islets of people with T2D [33]. Loss of one PDX1 allele in mice was shown to be sufficient to impair glucose tolerance already in young animals, prevented beta cell expansion during aging, and increased spontaneous apoptosis in islets when cultured at physiological glucose levels *ex vivo* [31,34]. We asked if P53 ablation improves glucose tolerance and beta cell function in PDX1^wt/KO^ mice by generating P53^BKO^ additionally lacking one PDX1 allele (PDX1^wt/KO^ P53^BKO^ mice). PDX1 heterozygosity and ablation of P53 were confirmed via qPCR and immunoblotting of pancreatic islets (Figs. S2A–C). 4-week-old PDX1^wt/wt^ P53^BKO^ (used as a control with normal PDX1 expression), PDX1^wt/KO^ Ctrl, and PDX1^wt/KO^ P53^BKO^ littermates were fed a HFD for 16 weeks, since HFD accelerates beta cell dysfunction in this model [35]. While body weight was similar in all three groups (Figure 2A), we observed increased (non-fasted and fasted) blood glucose levels (Figure 2B,C and S2D), strongly impaired glucose tolerance (Figure 2D–F and S2E-G) and reduced glucose stimulated insulin secretion (Figure 2G–I) in mice lacking one PDX1 allele compared to PDX1 wildtype mice, validating this model. Nonetheless, ablation of P53 in beta cells did not prevent fasting and random-fed hyperglycemia and severe glucose intolerance, and also failed to increase insulin secretion during repeated tests (Figure 2B–I and S2D-G). Notably, insulin sensitivity tended to be increased in both groups lacking one PDX1 allele compared to PDX1^wt/wt^ P53^BKO^ mice, but was not further affected by P53 deficiency (Figs. S2H and I). Since depletion of beta cell P53 did not preserve glucose homeostasis of HFD-fed PDX1^wt/KO^ mice, we asked if beta cell counts would be altered by ablation of P53, as previously observed in a glucokinase hyperactivation mouse model [18]. Nonetheless, the number of pancreatic beta cells (as detected by Nkx6.1 staining [33], Fig. S3) and islet cells (Hoechst^positive^ cells in glucagon, insulin and Nkx6.1 positive area) and islet area of PDX1^wt/KO^ P53^BKO^ mice was comparable to PDX1^wt/KO^ Ctrl mice when normalized to either the number of all pancreatic cells, or pancreas area, or islet cell number by multicolor immunoflourescence staining (Fig. 2J, S2J-L and S3). Similarly, the mean islet area, and the number of proliferating beta cells (Nkx6.1 and Ki67 double positive cells) was unchanged (Figure 2K, L and Fig. S2M, N). When exposed to a normal diet, PDX1^wt/KO^ Ctrl mice and PDX1^wt/KO^ P53^BKO^ mice also showed indistinguishable weight gain, glucose tolerance, insulin secretion and insulin sensitivity (Fig. S4A-N). Gene expression analysis of pancreatic islets from all groups of animals revealed that ablation of one PDX1 allele was sufficient to reduce expression of its canonical target gene SLC2A2 (also known as glucose transporter 2, GLUT2) by approx. 70%, independent of P53 status (Fig. S4O). Notably, mRNA levels of the established P53 target gene and master regulator of beta cell apoptosis and proliferation, PHLDA3 [36], were significantly increased by ablation of one PDX1 allele, and this was completely prevented when P53 was co-ablated in beta cells, again demonstrating the efficacy of our P53 KO approach (Fig. S4O). Taken together, ablation of P53 failed to prevent beta cell dysfunction in a model of monogenetic diabetes (with or without HFD feeding).

### 3.3 Beta cell specific P53 ablation fails to prevent STZ-induced hyperglycemia

**Figure 3:**
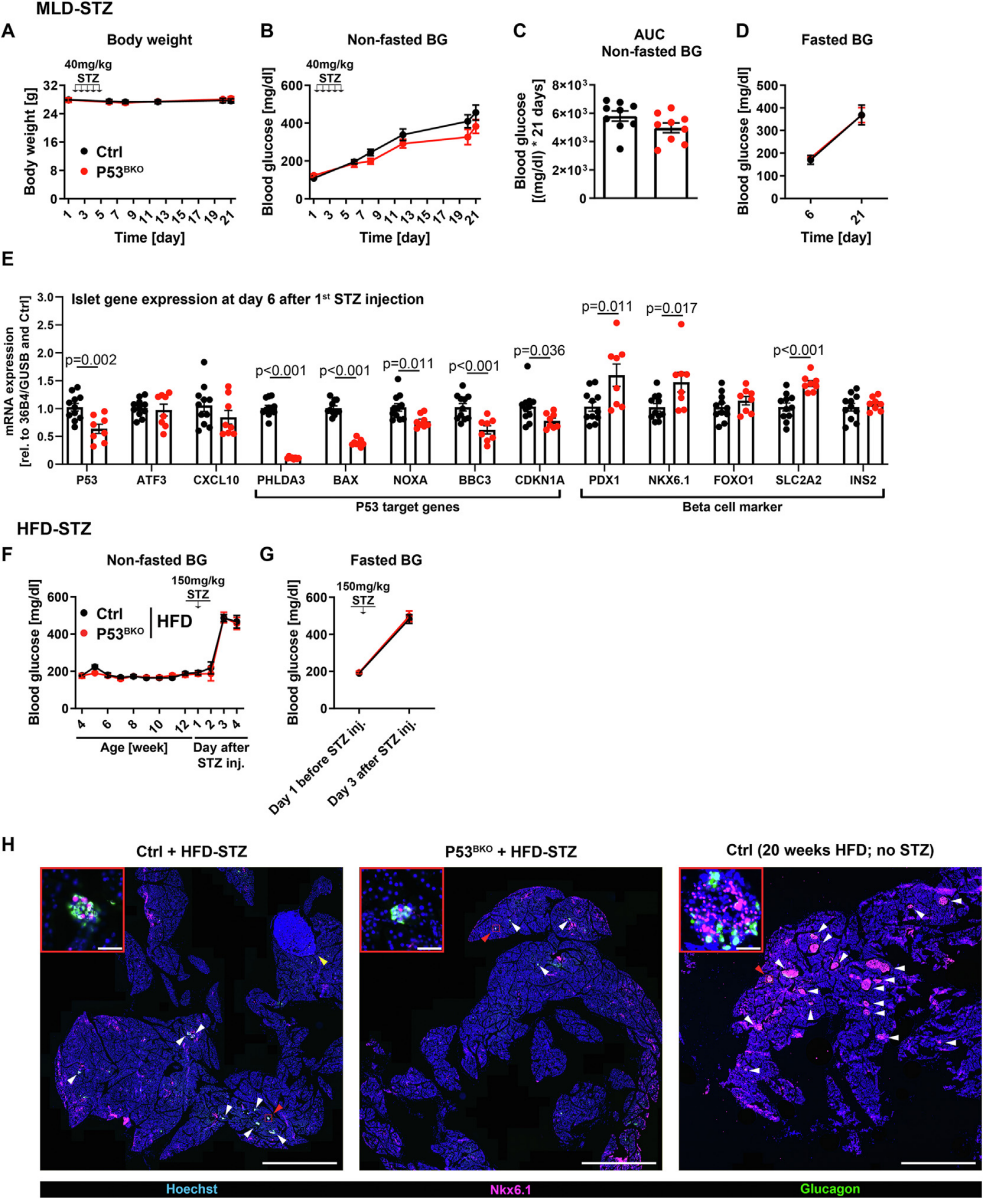
**STZ injection results in severe hyperglycemia independent of beta cell P53**. (**A**) Body weight (BW), (**B**) non-fasted blood glucose (BG) levels, (**C**) area under curve (AUC) of non-fasted BG levels and (**D**) fasted BG levels (6 h fasting) of Ctrl and P53^BKO^ mice injected five times with 40 mg streptozotocin (STZ) per kg BW on five consecutive days in week 12 of age (time point of first injection is stated as day 1). (**A**–**D**) Shown are mean and (**C**) individual values ± SEM (n = 2 independent cohorts with 9 Ctrl vs. 9 P53^BKO^ mice in total). (**E**) Relative islet mRNA expression levels of indicated genes of Ctrl and P53^BKO^ mice at day 6 after first STZ injection. Expression levels were normalized to the housekeeping genes *GUSB* and *36B4*, Ctrl was set to 1. Shown are mean and individual values ± SEM (n = 2 independent cohorts with 11 Ctrl vs. 8 P53^BKO^ mice in total). (**F**) Non-fasted and (**G**) 6 h fasted BG levels of HFD-fed Ctrl and P53^BKO^ mice injected once with 150 mg streptozotocin (STZ) per kg BW in week 12 of age (time of injection is stated as day 1). Shown are mean values ± SEM (n = 2 independent cohorts with 12 Ctrl vs. 11 P53^BKO^ mice in total). (**H**) Representative images of pancreatic sections of mice described in (F + G), as well as HFD-fed Ctrl mice without STZ injection (see Fig. 1), stained for nuclei with Hoechst (blue), beta cells with Nkx6.1 (pink) and alpha cells with glucagon (green). Shown are Nkx6.1 and glucagon positive islets (white arrows) and pancreatic lymph nodes (yellow arrow) (scale bar = 2 mm) and magnified insets of single islets indicated by red boxes (scale bar = 50 μm). Note the massive loss of Nkx6.1^positive^ beta cells after HFD + STZ treatment (for clear visibility, image contrast settings were optimized individually for separately stained HFD-STZ and HFD sections). Significance was determined by (**A** + **B**, **D**, **F** + **G**) two-way ANOVA followed by Sidak's multiple comparison test, (**C**) an unpaired two-sided student's t-test, or (**E**) multiple t-test's without correction for multiple comparisons.

We next asked, if P53 ablation might protect beta cells from cell death induced by a strong and acute pro-apoptotic stimulus. To induce beta cell death *in vivo*, we used the beta cell toxin STZ that enters beta cells via GLUT2, induces DNA fragmentation and accordingly activates the DNA damage response (DDR) including ATM [20,30,31,37,38]. When using the multiple low-dose STZ (MLD-STZ) model [8], hyperglycemia manifests within 2–3 weeks. In our study, we injected 40 mg/kg STZ on 5 consecutive days and measured blood glucose levels and body weight over the following 16 days (Fig. S5A). We found no differences in body weight or STZ mediated increase of blood glucose levels between P53^BKO^ and Ctrl mice (Figure 3A–D). We reasoned that potentially, activation of the DDR and a subsequent increase of pro-apoptotic mRNAs was mediated by proteins separate from P53, such as ATM [20]. However, we determined that mRNA levels of pro-apoptotic P53 target genes such as *PHLDA3*, *BAX*, *NOXA* and *BBC3*, as well as the cell cycle inhibitor *CDKN1A* were reduced by 25–90% in isolated islets from STZ treated P53^BKO^ mice compared to islets from Ctrl mice one day after the last STZ injection (Fig. 3E and S5B). Of note, *BAX*, *NOXA* and *BBC3* expression was not altered in islets from untreated P53^BKO^ mice (Fig. S4O), confirming that STZ treatment increases these apoptotic mRNAs in a P53 dependent manner. Expression of the ATM regulated T-cell chemoattractant CXCL10 [20], which was implicated in islet destruction, was similar between Ctrl and P53^BKO^ islets (Fig. 3E). In addition, expression levels of beta cell markers were stable or even increased (Fig. 3E). Thus, although islet cells from P53^BKO^ mice are protected against STZ-induced upregulation of pro-apoptotic P53 target genes, beta cell specific P53 depletion is insufficient to prevent MLD-STZ induced hyperglycemia.

We next tested if P53 ablation would protect beta cells and prevent diabetes acutely after extreme beta cell stress. To this end, we injected HFD-fed Ctrl and P53^BKO^ mice with one single high dose of STZ (HFD-STZ model, Figs. S5C–G) [8]. Strikingly, both P53^BKO^ and Ctrl mice manifested diabetes within 2 days after STZ injection (Figure 3F,G). Accordingly, nearly all (Nkx6.1^positive^) beta cells were lost 3 days after STZ injection independent of genotype (Fig. 3H). We conclude that depletion of P53 does not protect beta cells against STZ induced cell death and subsequent diabetes.

### 3.4 P53 ablation does not protect against Cytokine- or STZ-induced beta cell death *ex vivo*

**Figure 4:**
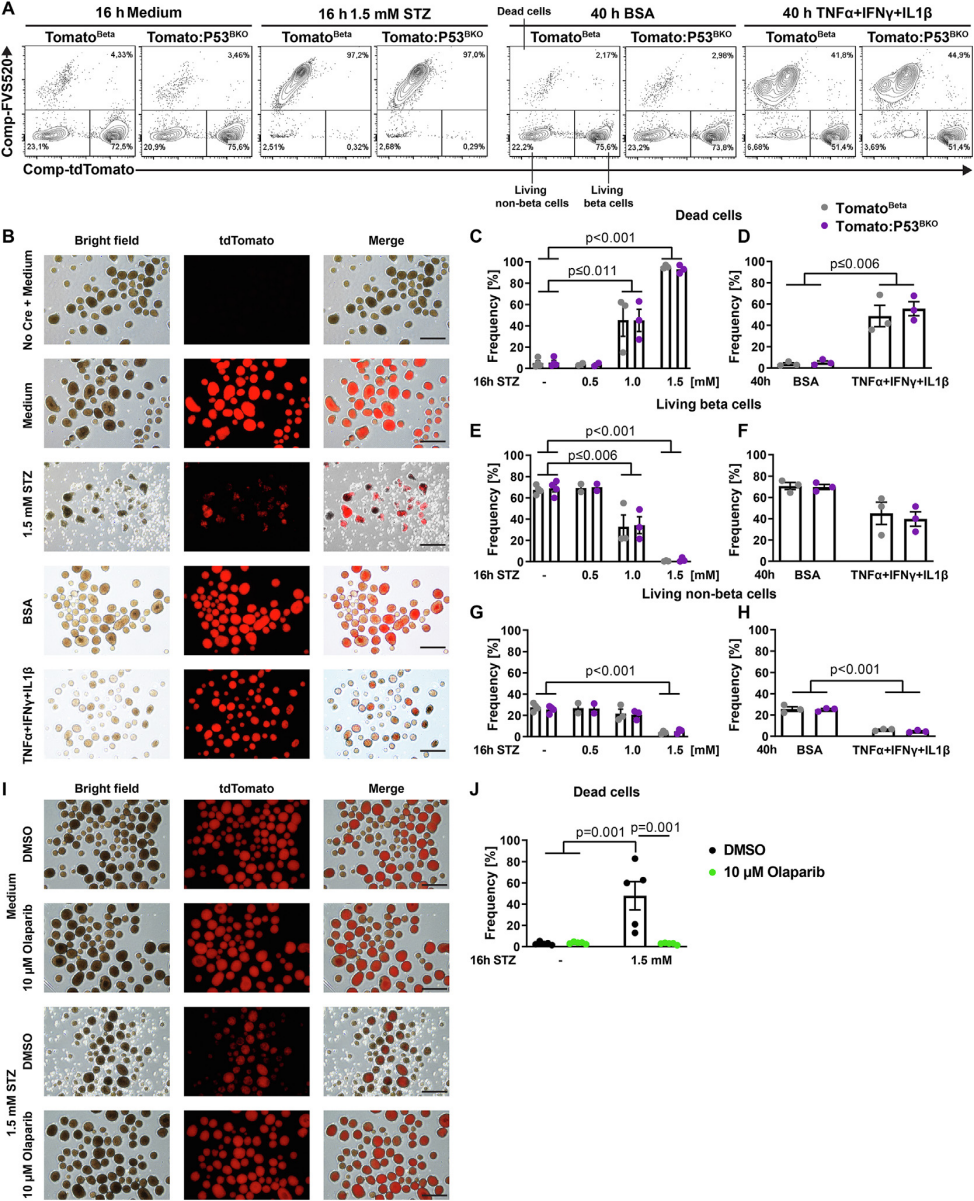
**PARP1 inhibition but not P53 ablation protects against STZ treatment *ex vivo***. (**A**) Representative flow cytometric analysis showing the gating strategy to detect three different cell populations: dead cells (FVS520^positive^), living beta cells (FVS520^negative^ + tdTomato^positive^) and living non-beta cells (FVS520^negative^ + tdTomato^negative^). Pre-gating strategy and representative analyses of additional conditions are shown in Figure S6. (**B**) Representative images of islets from Cre-negative and Tomato^Beta^ mice treated with a cytokine mix (TNFα+IL1β+IFNγ), STZ, or respective controls (pure medium or medium with BSA) depicting bright field, tdTomato (red) and merged channels (scale bar = 500 μm). (**C**–**H**) Frequency of (**C** + **D**) dead cells, (**E** + **F**) living beta cells and (**G** + **H**) living non-beta cells was determined in islets of Tomato^Beta^ and Tomato:P53^BKO^ mice after treatment with cytokine mix or STZ by flow cytometry as shown in (A). Shown are mean and individual values ± SEM (n = 3 (cytokine mix) and n = 2–4 (STZ) independent experiments; one dot represents pooled islets of 3–4 mice). (**I** + **J**) Pancreatic islets were pre-treated for 3 h with 10 μM Olaparib and for 16 h with Olaparib and 1.5 mM STZ or respective controls (DMSO and pure medium). (**I**) Representative images of one experiment with Tomato^Beta^ islets depicting bright field, tdTomato (red) and merged channels (scale bar = 500 μm). (**J**) Frequency of (FVS520^positive^) dead cells was determined via flow cytometry. Shown are mean and individual values ± SEM (n = 5 independent experiments with pooled islets of 4–10 mice per experiment). Significance was determined by two-way ANOVA followed by Sidak's multiple comparison test. (**C**, **E**, **G**) Significant comparisons between different STZ concentrations are not shown to ensure readability.

While our results so far indicated that P53 ablation is not able to prevent beta cell dysfunction or loss of beta cells, we wanted to directly quantify beta cell and islet cell death in a controlled *ex vivo* setting. To distinguish between beta and non-beta cells, we used P53^BKO^ and Ctrl mice additionally carrying a Cre-inducible reporter gene in the Rosa26 locus, resulting in a beta cell specific expression of a red fluorescent protein (tdTomato). Islets of these Tomato^Beta^ and Tomato:P53^BKO^ mice were isolated and treated with a pro-inflammatory cytokine mix (consisting of TNF-α, IL1-β and IFN-γ) widely used to stimulate beta cell death [39], or STZ. The bright viability stain FVS520 was used to identify dead (FVS520^positive^) and living (FVS520^negative^) cells by flow cytometry. Since the tdTomato signal disappeared in dying cells, most likely due to membrane leakiness, we quantified the percentage of living beta (tdTomato^positive^ and FVS520^negative^) cells, living non-beta (tdTomato^negative^, FVS520^negative^) cells, and dead (FVS520^positive^) cells (Fig. 4A and S6A-H). 1 mM STZ treatment for 16 h specifically reduced the living beta cell population, while incubation with 1.5 mM STZ caused nearly all islet cells to die (Figure 4B,C, E, G). In contrast, Cytokine incubation for 40 h resulted in 45–50% islet cell death, with both beta cells and interestingly, non-beta cells succumbing to the proinflammatory stimulus (Figure 4B,D, F, H). In line with our observations *in vivo*, islets of Tomato:P53^BKO^ mice were neither protected against Cytokine- nor STZ-induced cell death (Figure 4C–H). Similarly, islets from mice with beta cell specific ablation of ATM, an important DNA damage sensor and upstream regulator of P53 [16]), were not protected against STZ-induced cell death (Figs. S7A–H). To ensure that we would be able to detect a protective effect against STZ-induced beta cell death in our experimental setup, we tested the Poly (ADP-Ribose) Polymerase 1 (PARP1) inhibitor Olaparib, since PARP1 KO mice are strongly protected against STZ-induced diabetes [40]. Moreover, PARP1 and P53 directly or indirectly interact in other cell types [[41], [42], [43]]. Indeed, Olaparib was able to completely prevent STZ-induced cell death in isolated islets (Figure 4I,J) without preventing activation of the early cellular response to DNA damage (phosphorylation of Histone H2A.X, Figure S7I and J). Accordingly, our findings suggest that PARP1-induced cell death after STZ treatment in pancreatic beta cells is not dependent on ATM or P53 signaling. Overall, we show that ablation of P53 fails to protect against two major cell stress stimuli linked to beta cell loss in T1D and T2D (inflammation and DNA damage) [18,20,44].

## 4 Discussion and conclusion

Pro-apoptotic processes appear to be critical for dysfunction and loss of pancreatic beta cells observed in T1D, T2D and monogenetic forms of diabetes [9,14,35,45,46]. P53 is arguably the most important cellular regulator of cell death, and activation of P53 as well as other DDR proteins has been correlated to development of beta cell failure and loss [8,18,20]. While the functional role of P53 has mainly been studied in whole body (conventional) KO mice, or by pharmacologically induced systemic inhibition or activation in models of diabetes, comprehensive studies on the beta cell specific role of P53 *in vivo* have been missing. To address this, we analyzed the phenotype of beta cell specific P53 KO mice in several complementary models of diabetes development. We note that the knock-in Ins1-Cre strain used in our studies is distinct from several older transgenic beta cell Cre driver strains, that appear to affect beta cell function independent of conditional alleles, in part due to unintentional expression of growth hormone (GH) from the inserted transgene [47,48]. Indeed, we confirmed that at least one inducible beta cell Cre driver strain with detectable GH expression was highly resistent against STZ-induced hyperglycemia (data not shown).

Overall, our experimental approaches allowed us to investigate the effect of low, intermediate, high and extreme levels of beta cell stress and impairment *in vivo* and *ex vivo*. In contrast to our expectations, our results robustly show that P53 in beta cells is not involved in the regulation of beta cell numbers or function in multiple complementary models of diabetes *in vivo*. It is not immediately apparent, why the observed strongly reduced expression of pro-apoptotic mRNAs such as *PHLDA3*, *BAX* and *BBC3* is insufficient to ameliorate beta cell death in P53^BKO^ mice. For example, conventional PHLDA3 KO mice demonstrate diminished islet cell death as well as increased islet size [36]. Our results are in line with the notion that several parallel cellular pathways, with P53 being one of them, are activated during beta cell stress, which may underlie the sparse success of existing beta cell protective drugs to decisively reverse loss of insulin secretion capacity and diabetes progression in the clinical setting. Our findings are also along the line that P53 regulated pathways and mechanisms in cell types apart from beta cells mediate its effects on systemic physiology and glucose metabolism, such as endothelial cells [49].

Fluorescent reporter protein expression also allowed us to directly assess cell death in beta cell and non-beta cell compartments in pancreatic islets. We hereby directly demonstrated that the cytokine mix of TNF-α, IL1-β and IFN-γ that is widely used to induce beta cell death, as well as high dose STZ treatment, induces cell death of beta cells, but surprisingly also of non-beta cells. Future studies are needed to replicate this finding and identify the islet cell type that may be highly sensitive to inflammatory stress, since e.g. alpha cells are thought to be especially protected from Cytokine-mediated cell death [50]. In any case, since mechanistical studies on purported beta cell death in the context of T1D and T2D sometimes use methods not technically suited for cell type specific resolution of cell death (e.g. cleaved caspase immunoblots from whole islet extracts), our results clearly argue for use of more precise tools to quantify cell death.

As a limitation of our study, our experimental design allowed us to test multiple, but not all relevant or potentially occurring types of beta cell stress linked to development of diabetes. Hence, we point out that some (but potentially rare) types of beta cell stress may induce loss of beta cells in a P53-dependent manner, e.g. glucokinase hyperactivation or abnormal microRNA expression [8,18]. Beta cell loss may be further influenced by the specific genetic makeup of the mouse strains involved as well as experimental and environmental conditions (for example, exact diet composition and gut microbiome). Nonetheless, our studies markedly indicate that targeting canonical P53-dependent pro-apoptotic pathways appears to be an unfavourable approach for development of novel therapies for beta cell loss and diabetes. To conclude, our study reveals a negligible role of P53 in general beta cell failure and death *in vivo*.

## Author contribution

C. Uhlemeyer designed, performed, analyzed and interpreted most of the experiments with help by N. Müller, M. Rieck, T.F. Dorweiler, S. Heiduschka and K. Grieß. J. Kuboth and C. Schlegel provided technical assistance and performed some experiments. J. Eckel, M. Roden, E. Lammert and M. Stoffel provided scientific and experimental advice, methodology and mice. E. Lammert supervised C. Uhlemeyer and gave scientific advice. B.-F. Belgardt conceived and supervised the study, designed experiments, obtained funding and interpreted data. B.-F. Belgardt and C. Uhlemeyer wrote the manuscript. All authors read and commented on the manuscript and agreed on submission of the manuscript.

## Figures and Tables

**Figure:**
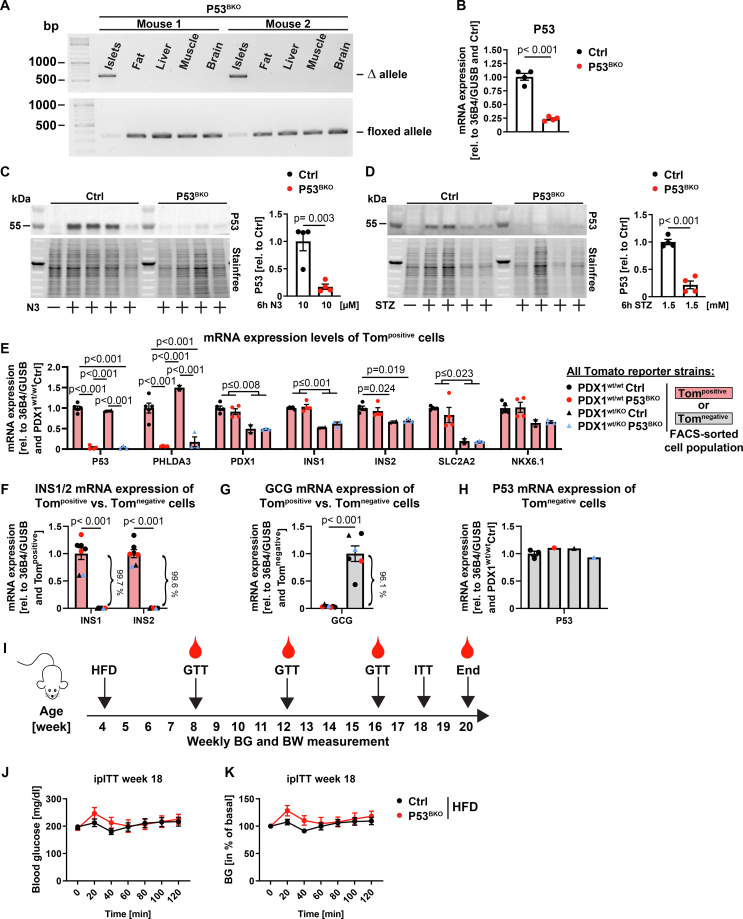
Fig. S1: Additional analyses of P53^BKO^ mice. (**A**) Gel-electrophoretic analysis of a P53 loxP and delta polymerase chain reaction (PCR) using DNA of different tissues from two individual P53^BKO^ mice as template. Presence of 612 bp delta amplicon indicates successful Cre driven recombination of the *P53* locus (exon 2-10 of the *P53* gene). Presence of 400 bp P53 floxed amplicon confirms loxP flanked *P53* allele. The color of the gel image was inverted and auto-adjusted in Image Lab (Bio-Rad). (**B**) Islet *P5*3 mRNA expression levels of Ctrl and P53^BKO^ mice. Expression levels were normalized to *GUSB* and *36B4*, levels of Ctrl mice were set to 1. Shown are mean and individual values ± SEM (n = 1 experiment with islets of 4 vs. 4 mice; one dot represents islets of one mouse). (**C** + **D**) Islet P53 protein levels (53 kDa) and quantifications of Ctrl and P53^BKO^ mice treated with (**C**) 10 μM Nutlin-3 (N3) (or DMSO) or (**D**) 1.5 mM STZ (or medium) for 6 h. Stainfree was used as loading control (n = 2 independent experiments with islets of 4 vs. 4 mice in total, one dot represents islets of one mouse). For quantification, P53 signal levels were normalized to stainfree levels. N3 or STZ treated Ctrls were set to 1. (**E**-**H**) Tom^positive^ and Tom^negative^ islet cells from PDX1^wt/wt^ Ctrl, PDX1^wt/wt^ P53^BKO^, PDX1^wt/KO^ Ctrl, and PDX1^wt/KO^ P53^BKO^ mice, each Ins1-Cre transgenic and also carrying a Cre-activatable tdTomato reporter allele, were FACS-sorted and used for qPCR analyses. Gating strategy is shown in Fig. S8. (**E**) mRNA expression levels of indicated genes of Tom^positive^ beta cells (n = 2 independent experiments with islets of 3–5 mice per group; one dot represents islets of 1–3 mice). (**F** + **G**) mRNA expression levels of *INS1*, *INS2* and *GCG* of Tom^positive^ vs. Tom^negative^ cells (n = 1 with islets of 2–3 mice per group; one dot represents islets of 1–3 mice). (**H**) *P5*3 mRNA levels of Tom^negative^ cells (n = 1 with islets of 1–3 mice per group; one dot represents islets of 1–3 mice). (**E**-**H**) Expression levels were normalized to *GUSB* and *36B4*, levels of (**E** + **H**) PDX1^wt/wt^ Ctrl mice, (**F**) Tom^positive^ or (**G**) Tom^negative^ cells were set to 1. (**I**) Schematic of the performed metabolic tests and interventions with HFD fed P53^BKO^ mice. (**J** + **K**) Glucose levels during an intraperitoneal insulin tolerance test (ipITT, 0.75 U insulin / kg BW) performed at the age of 18 weeks in mice described in Figure 1. (K) Basal BG levels were set to 100%. Significance was determined by (**B**-**D**, **G**) an unpaired two-sided student's t-test, (**F**) multiple t-test's without correction for multiple comparisons or (**E** + **H**) one-way or (**J** + **K**) two-way ANOVA followed by Tukey's or Sidak's multiple comparison test, respectively.

**Figure:**
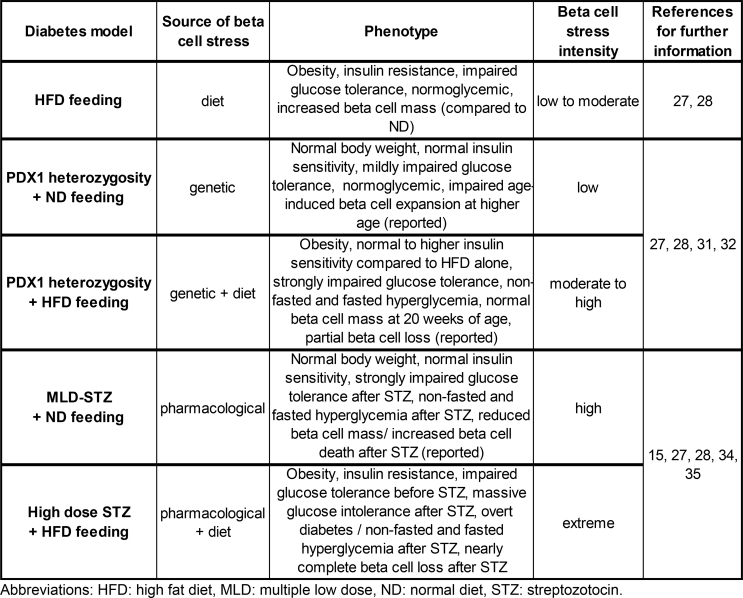
Table S1: Overview over diabetes models used in this study.

**Figure:**
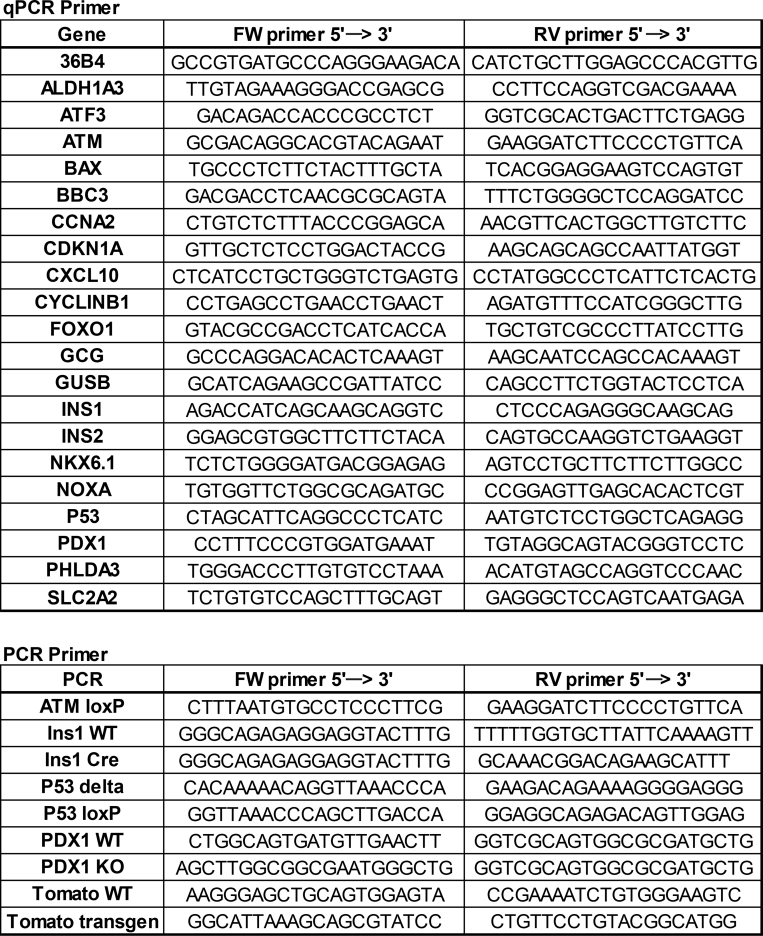
Table S2: Primer sequences. P53 in pancreatic beta cells

**Figure:**
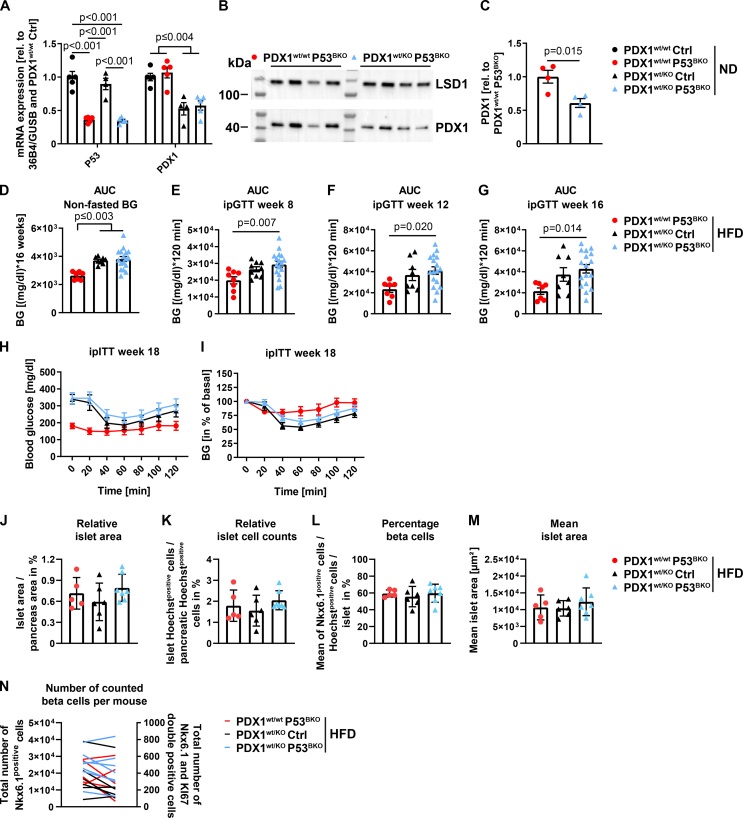
Fig. S2: Additional analyses of HFD fed PDX1^wt/KO^ P53^BKO^ mice. (**A**) *P53* and *PDX1* islet mRNA expression levels of 7–10 weeks old PDX1^wt/wt^ Ctrl, PDX1^wt/wt^ P53^BKO^, PDX1^wt/KO^ Ctrl and PDX1^wt/KO^ P53^BKO^ mice. Expression levels were normalized to *36B4* and *GUSB*, levels of PDX1^wt/wt^ Ctrl mice were set to 1. Shown are mean and individual values ± SEM (n = 3 independent experiments with 7–9 mice per group in total; one dot represents islets of 1–3 mice). (**B**) Islet PDX1 protein levels (42 kDa) of PDX1^wt/wt^ P53^BKO^ and PDX1^wt/KO^ P53^BKO^ mice. LSD1 (110 kDa) was used as loading control (n = 1 experiment with islets of 4 vs. 4 mice). (**C**) Quantification of western blot shown in (B) after normalization to LSD1 signal levels. Levels of PDX1^wt/wt^ P53^BKO^ were set to 1. (**D**-**G**) Area under curve (AUC) of (**D**) non-fasted BG levels and (**E**-**G**) BG levels for ipGTTs described in (Figure 2D–F). (**H** + **I**) Glucose levels during an intraperitoneal insulin tolerance test (ipITT, 0.75 U insulin / kg BW) performed in mice described in (Fig 2) at the age of 18 weeks. (**I**) Basal BG levels were set to 100%. (**J**-**N**) Quantification of (**J**) islet area normalized to pancreatic area, (**K**) islet cell numbers normalized to pancreatic cell numbers, (**L**) percentage of (Nkx6.1^positive^) beta cells, (**M**) mean islet area and (**N**) total numbers of counted Nkx6.1^positive^ + Ki67^positive^ beta cells per mouse of PDX1^wt/wt^ P53^BKO^, PDX1^wt/KO^ Ctrl and PDX1^wt/KO^ P53^BKO^ mice (n = 12–18 sections from n = 5–7 mice per group). Significance was determined by (**C**) an unpaired two-sided student's t-test or (**A**, **D**-**G**, **J**-**M**) one-way or (**H** + **I**) two-way ANOVA followed by Tukey's or Sidak's multiple comparison test, respectively. (**H** + **I**) Significant comparisons vs. PDX1^wt/wt^ P53^BKO^ are not shown to maintain visual clarity.

**Figure:**
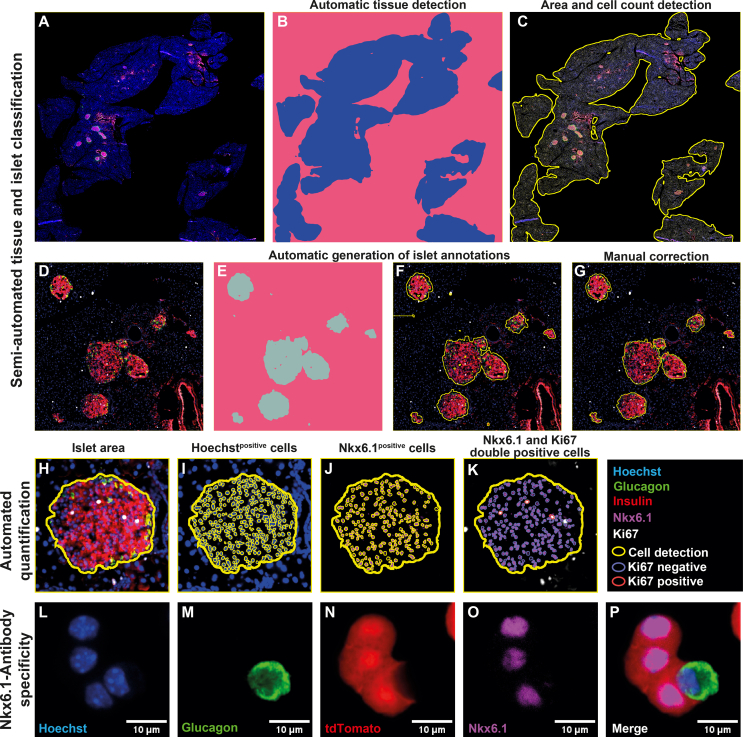
Fig. S3: Semi-automatic image analysis of pancreatic islets via QuPath. (**A**-**C**) Representative analysis of a pancreatic section including automatic generation of an annotation (by using a trained pixel classifier) allowing the quantification of tissue area and total (Hoechst^positive^) cell counts. (**D**-**E**) Pancreatic islets were automatically identified by insulin, glucagon and Nkx6.1 signal. (**F**-**G**) Single annotations were generated automatically and were manually adapted. (**H**–**K**) Of each islet, area and number of all (Hoechst^positive^) islet-, (Nkx6.1^positive^) beta- and (Nkx6.1 and Ki67 double positive) proliferating beta cells were automatically quantified and manually controlled. (**L**-**P**) Validation of nuclear staining of Nkx6.1, a beta cell specific transcription factor, by co-staining dispersed islet cells from Tomato^Beta^ mice with Hoechst and antibodies detecting Nkx6.1 and glucagon.

**Figure:**
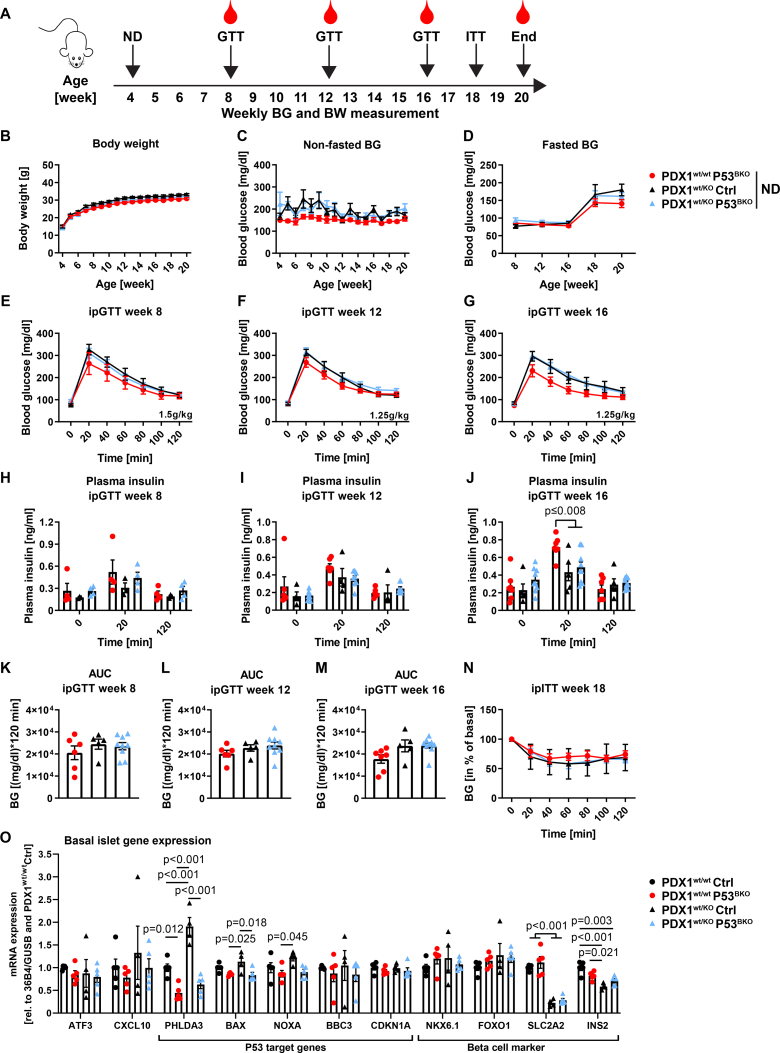
Fig. S4: PDX1^wt/KO^ mice show mild glucose intolerance independent of beta cell P53. (**A**) Schematic of the performed metabolic tests and interventions in normal diet (ND) fed PDX1^wt/wt^ P53^BKO^, PDX1^wt/KO^ Ctrl and PDX1^wt/KO^ P53^BKO^ mice. (**B**) Body weight, (**C**) non-fasted blood glucose (BG) levels and (**D**) fasted BG levels of PDX1^wt/wt^ P53^BKO^, PDX1^wt/KO^ Ctrl and PDX1^wt/KO^ P53^BKO^ mice fed a ND. Fasting period was 16 h (week 8, 12, 16), 4 h (week 18) and 6 h (week 20). (**E**-**G**) Blood glucose excursions during intraperitoneal glucose tolerance tests (ipGTT) at different ages of mice described in (A-D). Glucose bolus is indicated for each ipGTT. (**H**-**J**) Plasma insulin levels at time points 0, 20 and 120 min during ipGTTs described in (E-G). (**K**-**M**) Area under curve (AUC) of ipGTTs described in (E-G). (**N**) Glucose levels during an intraperitoneal insulin tolerance test (ipITT, 0.75 U insulin / kg BW) performed in mice described in (A-D) in week 18. Basal BG levels were set to 100%. (**B**–**N**) Shown are mean and (**H**-**M**) individual values ± SEM (n = 2 independent cohorts with 4–7 PDX1^wt/wt^ P53^BKO^ vs. 3-5 PDX1^wt/KO^ Ctrl vs. 4-9 PDX1^wt/KO^ P53^BKO^ mice in total). (**O**) Basal islet mRNA expression levels of indicated genes of 7–10 weeks old PDX1^wt/wt^ Ctrl, PDX1^wt/wt^ P53^BKO^, PDX1^wt/KO^ Ctrl and PDX1^wt/KO^ P53^BKO^ mice. Expression levels were normalized to the housekeeping genes *GUSB* and *36B4*, levels of PDX1^wt/wt^ Ctrl mice were set to 1. Shown are mean and individual values ± SEM (n = 3 independent experiments with 7–9 mice per group in total; one dot represents islets 1-3 mice). Significance was determined by (**B**-**J**, **N**) two-way or (**K**-**M**, **O**) one-way ANOVA followed by Sidak's or Tukey's multiple comparison test, respectively. Significant comparisons (**B**-**G**, **N**) vs PDX1^wt/wt^ P53^BKO^ or (**H**-**J**) between different time points are not shown to ensure readability.

**Figure:**
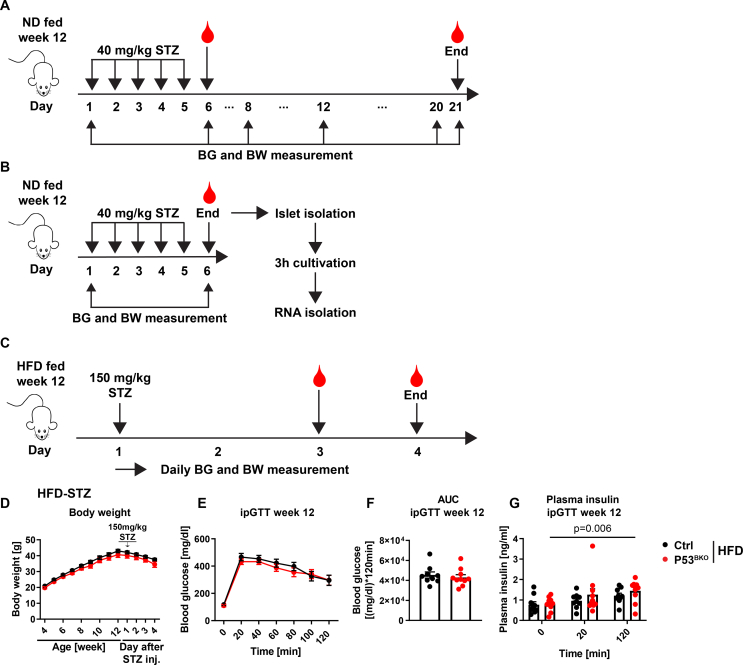
Fig. S5: Additional analyses for STZ *in vivo* experiments. (**A**) Schematic for MLD-STZ analyses. (**B**) Schematic for MLD-STZ approach used for islet isolations. (**C**) Schematic for HFD-STZ analyses. (**D**) Body weight (BW) of mice described in Fig 3F–H. (**E**) BG levels during ipGTT (1.5 g d-glucose in PBS / kg BW) performed in week 12 of age before STZ injection in HFD-STZ mice (n = 2 independent cohorts with 9 Ctrl vs. 10 P53^BKO^ mice in total). (**F**) Area under curve (AUC) and (**G**) plasma insulin levels (measured by ELISA) at time points 0, 20 and 120 min of the ipGTT described in (E). (**D**-**G**) Shown are mean and (**F** + **G**) individual values ± SEM. Significance was determined by (**D**, **E**, **G**) two-way ANOVA followed by Sidak's multiple comparison test, or (**F**) an unpaired two-sided student's t-test.

**Figure:**
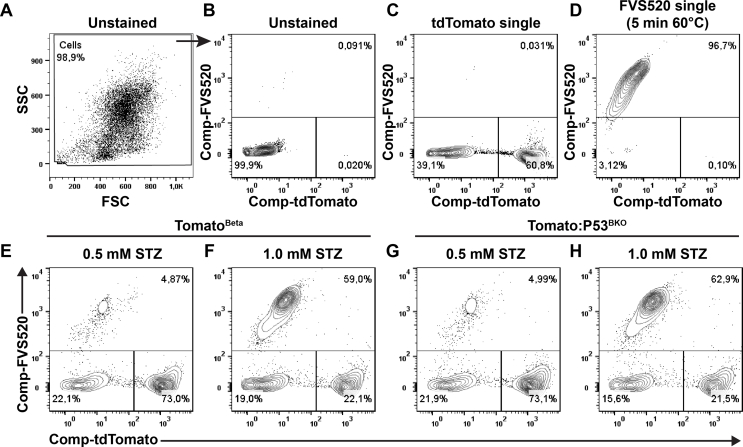
Fig. S6: Flow cytometry gating strategy and additional STZ experiments. (**A**) FSC vs. SSC of unstained (Cre-negative) islet cells. “Cells”-gate was used for exclusion of cell debris prior to further analyses. (**B**-**D**) Compensated FVS520 vs. tdTomato intensity of (**B**) unstained (Cre-negative) cells, (**C**) unstained Tomato^Beta^ cells and (**D**) (Cre-negative) cells stained with FVS520 (cells were killed by 60 °C heat shock for 5 min as positive control). (**E-H**) Compensated FVS520 vs. tdTomato intensity of 0.5 or 1 mM STZ treated Tomato^Beta^ or Tomato^:^P53^BKO^ islet cells, as indicated (depicted are representative analyses of additional conditions relevant to Fig. 4) (n = 1 representative experiment out of 2–4 independent experiments with a total of 12 vs. 14 mice; for each experiment, islets of 3–4 mice per genotype were pooled).

**Figure:**
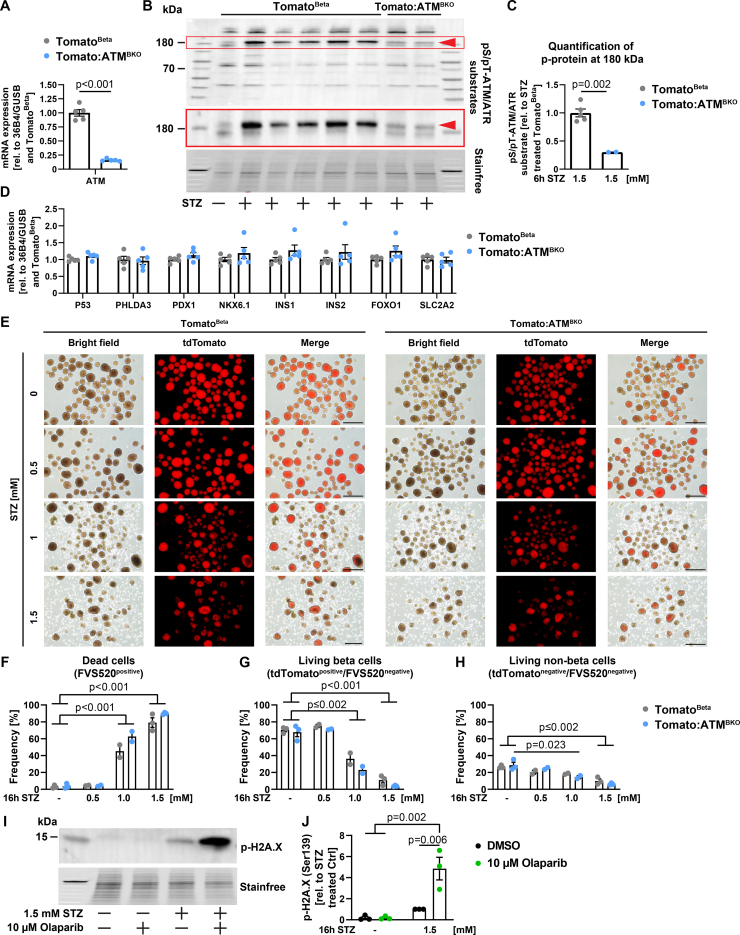
Fig. S7: ATM ablation does not protect against STZ-induced cell death *ex vivo*. (**A**) *ATM* islet mRNA expression levels of Tomato^Beta^ and Tomato:ATM^BKO^ mice. Expression levels were normalized to *GUSB* and *36B4*, levels of Tomato^Beta^ mice were set to 1. Shown are mean and individual values ± SEM (n = 1 experiment with islets of 5 vs. 5 mice; one dot represents islets of one mouse). (**B**) Relative protein amount of pS/pT-ATM/ATR substrates of Tomato^Beta^ and Tomato:ATM^BKO^ islets treated for 6 h with 1.5 mM STZ or medium as control. Stainfree signal was used as loading control. Blots were compressed in height. Area in red box is additionally shown uncompressed below. (**C**) Quantification of STZ induced phosphorylated protein (single band at 180 kDa, indicated by red arrow) after normalization to stainfree signal levels. Levels of STZ treated Tomato^Beta^ were set to 1 (n = 1 experiment with islets of 5 vs. 2 mice, one dot represents islets of one mouse). Note that STZ-induced phosphorylation of a protein at approximately the same size was also reduced by inhibition of ATM *in vitro* [37]. (**D**) Islet mRNA expression levels of indicated genes of same islets as described in (A). (**E**) Representative images of Tomato^Beta^ and Tomato:ATM^BKO^ islets treated with 0.5, 1 or 1.5 mM STZ (or medium as control) depicting bright field, tdTomato (red) and merged channels (scale bar = 500 μm). (**F**–**H**) Frequency of (**F**) dead cells, (**G**) living beta cells and (**H**) living non-beta cells was determined in islets of Tomato^Beta^ and Tomato:ATM^BKO^ mice after treatment with STZ by flow cytometry (as described in Fig. 4). Shown are mean and individual values ± SEM (n = 2–3 independent experiments with islets of 10 vs. 5 mice in total; one dot represents islets of 1–5 mice). (**I**) Relative protein amount of the DNA damage marker pS139-H2A.X in islets after STZ and/or Olaparib treatment as described in Figure 4J. Stainfree was used as loading control (n = 3 independent experiments with pooled islets of 7–8 mice). Stainfree blot was compressed in height. Note that Olaparib further increases the STZ-induced pS139-H2A.X signal as an indicator of unrepaired DNA damage, likely due to preventing PARP1-mediated DNA repair and consequently DNA repair-mediated NAD^+^-depletion (see main text). (**J**) Quantification of western blot shown in (**I**) after normalization to Stainfree signal levels. Levels of STZ and DMSO treated islets were set to 1. Significance was determined by (**A**, **C**) an unpaired two-sided student's t-test, (**D**) multiple t-test's without correction for multiple comparisons or (**F**–**H**, **J**) two-way ANOVA followed by Sidak's multiple comparison test, respectively. (**F**–**H**) Significant comparisons between different STZ concentrations are not shown to ensure readability.

**Figure:**
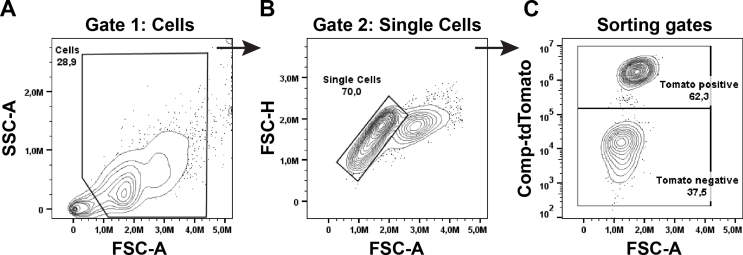
Fig. S8: FACS-gating strategy for isolation of beta- and non-beta cells. (**A**) FSC-A vs. SSC-A of an islet cell population of a representative Tomato reporter mouse for analyses in Fig. S1E-H. “Cells”-gate was used for exclusion of cell debris prior to further analyses. (**B**) FSC-A vs. FSC-H of the “Cells”-population described in (A). “Single Cells”-gate was used for exclusion of cell doublets prior to further analyses. (**C**) FSC-A vs. tdTomato of the “Single Cells”-population described in (B). “Tomato positive” and “Tomato negative”-gates were used for cell sorting of beta- and non-beta cells, respectively.

**Figure:**
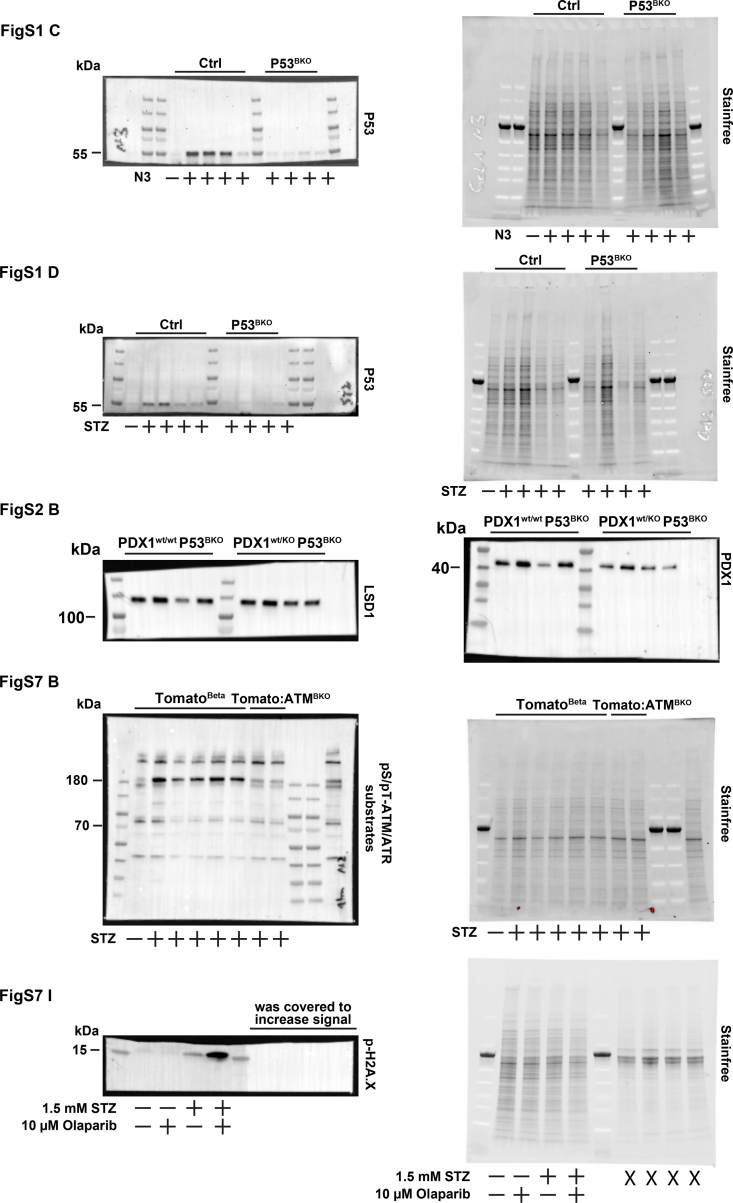
Fig. S9: Source data for immunoblot images. Uncropped blots for blots depicted in indicated figures.

## Data Availability

Data will be made available on request.
